# Divergence in brain size and brain region volumes across wild guppy populations

**DOI:** 10.1098/rspb.2021.2784

**Published:** 2022-08-31

**Authors:** Angie S. Reyes, Amaury Bittar, Laura C. Ávila, Catalina Botia, Natalia P. Esmeral, Natasha I. Bloch

**Affiliations:** Department of Biomedical Engineering, University of Los Andes, Bogota, Colombia

**Keywords:** brain evolution, sexual dimorphism, guppy, sexual selection, neuroanatomy

## Abstract

Complex evolutionary dynamics have produced extensive variation in brain anatomy in the animal world. In guppies, *Poecilia reticulata*, brain size and anatomy have been extensively studied in the laboratory contributing to our understanding of brain evolution and the cognitive advantages that arise with brain anatomical variation. However, it is unclear whether these laboratory results can be translated to natural populations. Here, we study brain neuroanatomy and its relationship with sexual traits across 18 wild guppy populations in diverse environments. We found extensive variation in female and male relative brain size and brain region volumes across populations in different environment types and with varying degrees of predation risk. In contrast with laboratory studies, we found differences in allometric scaling of brain regions, leading to variation in brain region proportions across populations. Finally, we found an association between sexual traits, mainly the area of black patches and tail length, and brain size. Our results suggest differences in ecological conditions and sexual traits are associated with differences in brain size and brain regions volumes in the wild, as well as sexual dimorphisms in the brain's neuroanatomy.

## 1. Introduction

Brain size is intimately related to functional and cognitive abilities [1,2] critical to an animals' fitness and survival. These selective pressures are responsible for the extensive adaptive variation in brain size we see across vertebrates. The brain, however, is a slow-growing and costly tissue, which imposes complex trade-offs with other tissues as contemplated under the expensive tissue hypothesis [3,4]. This hypothesis entails the costs associated with brain growth are only justified when there is sufficient selection for increased cognitive abilities and other brain functions [1,5,6], as shown across different taxa [3,7–11].

Larger relative brain size is associated with enhanced cognitive abilities that can evolve in response to various ecological pressures. The environment could impose cognitive challenges and select for behaviours that would ultimately impact brain size due to adaptation or plasticity [12–17]. We know habitat complexity [18–20], foraging strategies and other environmental factors can be strong drivers of brain size [21–23]. Furthermore, selection on the brain can often be domain-specific, impacting the relative volumes of different brain regions, and thus the brain's neuroanatomy [8,24]. We would thus expect to see pervasive variation in brain size and neuroanatomy across natural populations in different environments, particularly in brain regions associated with cognition.

Among the multiple biotic and abiotic factors that can impact the brain's neuroanatomy, predation has received particular attention [25–27]. Predator-related performance could improve with brain size due to a larger investment in cognitive, sensory and motor abilities to evade predators, selecting for a larger brain [27–29]. However, predation has also been reported to be negatively associated with brain size, likely in response to alternative predation strategies. The relationship between predation and brain evolution is therefore complex and may be mediated by many environmental and social factors [25,30], as well as differences in evasion tactics between sexes [31–34].

Ecological and social pressures, as well as predation, can impose cognitive demands that impact brain development and result in variation in the scaling of brain regions. Decades of research on brain divergence in the face of these changing selective pressures have resulted in two alternative models of brain evolution. On the one hand, the ‘mosaic’ evolution model posits functional pressures on different brain regions will lead to region-specific scaling factors [35–38]. By contrast, the ‘concerted’ evolution hypothesis posits brain region proportions are constrained by developmental programs [39–41]. This remains a debated issue in evolutionary neuroscience as evidence has accumulated in favour of both evolutionary models [42]. In this regard, intraspecific comparative studies across wild populations can be a powerful approach to better understand brain evolution [43,44].

The guppy, *Poecilia reticulata*, is a small livebearing fish and a long-standing model in evolutionary biology and sexual selection [45]. Guppies can successfully colonize diverse fresh-water environments with widely different ecological conditions [38,45]. Male guppies are highly variable in their nuptial colours and other sexual traits, and this variation has been linked to varying ecological and predation pressures as well as female preferences [26,46,47].

A series of recent laboratory studies based on individuals selected for large or small relative brain size [26,28,48,49] have significantly contributed to our understanding of brain evolution, allowing us to formulate hypotheses that could be tested in the wild. Guppies from large brain selection lines performed better in cognitive tasks [8,50], differed in their anti-predator behaviour and showed improved survival [27,28]. On the other hand, a larger brain was also associated with a reduced innate immune response, smaller guts, and decreased offspring production [48,51,52]. Interestingly, it was also shown that brain size is associated with nuptial traits expression, suggesting a genetic association between brain size and guppy nuptial traits [53].

The association between sexual traits and brain size reported in laboratory guppies supports the general hypothesis that the cognitive demands imposed by competition for mates and mate selection drive an increase in brain size [54–57]. Hypotheses on the balance of sexual and natural selection for brain evolution, however, are still controversial and we have yet to determine whether the relationship between sexual traits and brain size is maintained under changing environmental conditions in the wild [58]. Previous studies have also reported the growth of specific brain regions in response to mating and courting selective pressures [54–57]. In guppies, multiple nuclei involved in mating processes lie in the telencephalon [37,59], and we thus expect to find an association between sexual traits and the volume of the telencephalon across natural populations.

In Colombia, guppies have diversified across multiple river systems (Magdalena-Cauca, Amazonas y Pacífico) and lakes since the 1930s [60], and more recently after a series of introductions for mosquito-borne disease control [61]. Guppies are present across the country in very diverse environments making this an ideal system to test our hypotheses on brain divergence. We collected guppies across 18 populations in this previously unexplored system to evaluate the extent of brain differentiation across natural populations, and how it is related to variation in habitat characteristics, predation and sexual traits. Overall, our study allows us to inquire about the mechanisms at the basis of divergence in brain size and neuroanatomy to better understand its evolution.

## 2. Material and methods

### (a) Sample collection

We sampled 18 populations in diverse environments across Colombia (electronic supplementary material, figure S1), collecting 10 females and 10 males per population for a total of 360 individuals. Populations can be classified into three categories: lentic (lake or still water), lotic (river or stream) and intervened (human-built environments like fisheries or small artificial lakes). Locations and details for each population can be found in the electronic supplementary material, tables S1 and S2. Body length for each individual was measured in the field (electronic supplementary material, table S3), and photographs were taken from both sides of each fish next to a colour reference (X-rite colour checker) and a ruler for later analysis. Individual heads were preserved with 4% formaldehyde and transported to the laboratory for detailed dissections under a stereoscope (Olympus SZX7) and neuroanatomical measurements. More details can be found in the electronic supplementary material.

### (b) Sexual trait quantification

Female guppies mainly select males based on gonopodium length, the length of the caudal fin, as well as the area of colour patches and the saturation of orange patches [53,62]. We estimated the total areas of colour patches for each individual using ImageJ [63], averaging measurements both sides of the fish and orange saturation using Just Colour Picker (Version 5.5. (2020), Annystudio). Orange saturation was normalized to the orange reference for proper interindividual comparisons. The caudal fin was measured from the end of the caudal peduncle to the tip of the middle tail ray and referred to as tail length throughout. Gonopodium length was measured from its base to the tip of the last fin ray.

### (c) Brain dissection and neuroanatomy

Brains were weighed to the nearest 0.001 mg in an analytical balance (VIBRA HT 224RCE) following standard protocols in the literature [26]. All dissections and weight measurements were performed by one person (ASR) and in random order to avoid any biases. We then measured five brain regions, the telencephalon, optic tectum, cerebellum, hypothalamus and dorsal medulla for all individuals (electronic supplementary material, figures S2 and S3). Measurements were done blind to the population of origin. Here, we photographed brains in lateral, dorsal and ventral views (Olympus SZX7 stereoscope coupled to an Axiocam ERc 5 s camera). Photographs were then used to measure the height, length and width for each region in ImageJ [26], with very high repeatability for all brain region measurements (*r* = 0.92–0.97; *p* < 0.01) [17,64].

### (d) Predation pressure estimation

As a rough estimate of predation risk, we considered the presence/absence of major guppy predators at the different sampling sites. Each population was assigned a predation score from 0 to 5 according to the number of predators that are present at each location. We used bibliographic references, local observations and inquiries with local communities for these estimates (electronic supplementary material, tables S4A and S4B).

### (e) Statistical analyses

#### (i) Differences in whole brain size across populations

We used a linear model to examine variation in brain weight across populations and between sexes, as well as its association with environment type and predation pressures, included as fixed effects, while controlling for body length as covariate. All continuous variables were log-transformed and analyses were done using lme4 (v. 1.1–13) [65] and lmerTest (version 2.0–33) packages in R.

#### (ii) Brain region volumes

Here, we performed a similar series of linear models to examine the variation in the volumes of each of the five brain region volumes measured, relative to populations, sex, environment type and predation pressure. In these models, whole brain weight was included as a covariate. All continuous variables were log-transformed.

We also investigated the allometric relationship of each brain region relative to brain weight, and whether this relationship changed across populations. To do this, we tested for significant differences in the slope and intercept of the regressions across populations following the same methodology used by Santini & Isaac [66]. We used a series of nested models for each brain region against brain weight (allometric power law: log(BR) − log(a) + b.log(BW)) across populations for each sex separately as follows: (i) To assess whether the intercept of the regression of brain region on brain weight changes across populations, we tested for the effect of population using a model that only includes population as a fixed effect. (ii) To assess for differences in the slope, we used a similar model that included the interaction between population and brain weight. Finally, to evaluate the simultaneous change of the intercept and slope, we included the population as well as the previous interaction in the model. All models were compared using the Akaike information criterion (AIC).

#### (iii) Relationship between brain characteristics and sexual traits

In order to evaluate how sexual traits are associated with relative brain weight and brain region volumes, we started by testing for covariation between the sexual traits using a principal component analysis. Scores from the significant principal components were then extracted for downstream statistical analysis. These models were done using scores for the first three sexual traits PCs as dependent variables in three separate linear models that included brain weight, environment type, predation score as fixed effects, body length as a covariate and population as a random effect. To investigate the association between each of the sexual trait PCs and brain regions, volumes for each region were included in three separate models as fixed effects, together with environment type, predation, brain weight as covariate and population as a random effect.

### (f) Ang-1 relative expression

We collected additional brain samples in RNAlater for five individuals of each sex in order to estimate relative expression of Angiopoietin-1 (*Ang-1*) for four populations: LM, QM, LV and PA (see details in the electronic supplementary material, methods). For these samples, we extracted total RNA with Aurum Total RNA Mini Kit (BIO-RAD, USA), and reverse transcribed total RNA to obtain cDNA using random hexamers with cDNA using iScript Select cDNA Synthesis Kit (BIO-RAD, USA). We performed real-time PCR for *Ang-1* and two housekeeping genes, *β-actin* and *rpl13a*, with primers detailed in the electronic supplementary material, methods, using iTaq universal SYBR Green supermix kit (BIO-RAD, USA) in a Qiagen qRT-PCR ROTOR-GENE Q machine (Qiagen, USA). The resulting fluorescence values were used to obtain *Ang-1* expression values normalized to the average expression of the two housekeeping genes in DART-PCR (as detailed in the electronic supplementary material).

We initially performed an ANOVA with *Ang-1* to determine whether *Ang-1* relative expression was statistically different across populations and between sexes. We then used a mixed model to evaluate the relationship between *Ang-1* relative expression and brain size, including body length as a covariate and population as a random effect.

## 3. Results

### (a) Differences in brain weight across populations

**Figure 1.**
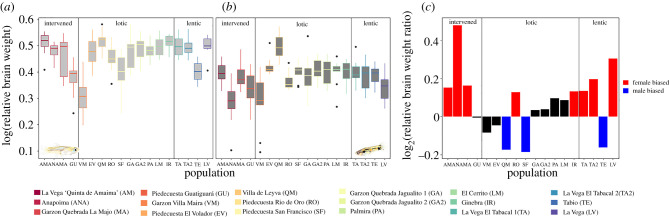
Differences in relative brain weight across populations, calculated as the ratio of brain weight to body length in (*a*) females and (*b*) males. (*c*) Differences in the degree and direction of sexual dimorphism in relative brain weight across populations (in log_2_ scale). Red bars with positive values correspond to populations in which relative brain weight is larger for females, blue bars with negative values to populations in which relative brain weight is larger for males and black bars to populations with no sexual dimorphism in relative brain weight using a fold-change threshold of 0.1. Populations are shown in the *x*-axis of each figure and vertical divisions in (*a*–*c*) indicate populations from the three types of environments: intervened, lotic and lentic. (Online version in colour.)

We found significant differences in relative brain weight across populations (*F*_10,287_ = 23.730; *p* < 0.001; electronic supplementary material, table S5), and between sexes (*F*_1,287_ = 636.983; *p* < 0.0001; electronic supplementary material, table S5; figure 1). It is worth noting that the direction of the sexual dimorphism in relative brain weight changes across populations (interaction between sex and population *F*_10,287_ = 3.269; *p* < 0.001; electronic supplementary material, table S5), with populations in which females have larger brains and others where this relationship is reversed (figure 1*c*). Moreover, we found significant differences in relative brain weight in association with the environment type and predation (environment type: *F*_2,287_ = 6.757; *p* < 0.01; electronic supplementary material, figure S4 and table S5. Predation: *F*_5,287_ = 40.033; *p* < 0.001; electronic supplementary material, figure S5 and table S5). As expected, the effects of predation pressure and environment type on relative brain weight change for males and females (interaction between sex and predation *F*_5,287_ = 3.409; *p* < 0.01; interaction between sex and environment type *F*_2,287_ = 4.752; *p* < 0.01; electronic supplementary material, figure S5 and table S5). However, these associations do not seem to be mediated by the type of predator present in each population (electronic supplementary material, table S6).

The mechanisms by which brain size changes have been previously associated with *Ang-1* expression in guppies and zebrafish [67]. We measured the relative expression of *Ang-1*, in four of our populations. Preliminary quantification of *Ang-1* expression showed no significant changes in the expression of this gene across populations or between sexes (population: *F*_3,20_ = 1.872; *p* = 0.19; sex: *F*_1,20_ = 0.232; *p* = 0.64; electronic supplementary material, figure S6 and table S7). Not surprisingly we found no significant association between *Ang-1* relative expression and brain weight in either sex (*p* > 0.3 for both males and females; electronic supplementary material, table S8)

### (b) Differences in relative brain region volumes

**Figure 2.**
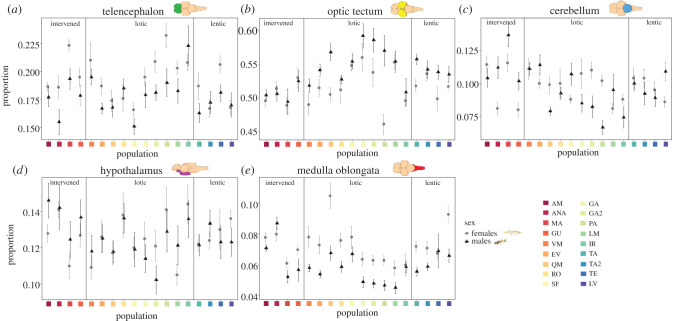
Differences in relative brain region volumes for males and females across populations (*a*) telencephalon, (*b*) optic tectum, (*c*) cerebellum, (*d*) hypothalamus and (*e*) medulla oblongata. Brain drawings in each graph highlight the corresponding brain region. The y-axis indicates the proportion of each region relative to the whole brain. The *x*-axis indicates the population and vertical divisions indicate the populations from the three types of environments: intervened, lotic and lentic. (Online version in colour.)

We used linear models meant to evaluate variation in relative brain regions volumes across populations and between sexes, and whether relative brain region volumes are changing in association with the environment type and predation pressures. We found highly significant differences in the relative volume of all brain regions across populations (all regions *p* < 0.0001, figure 2; electronic supplementary material, figure S7 and table S9), and between sexes (all regions *p* < 0.05, figure 2; electronic supplementary material, table S9). Interestingly, the optic tectum is the only brain region with male-biased sexual dimorphism, while females had larger relative volumes for all other regions (figure 2; electronic supplementary material, table S9). These results indicate that the differences we observe in relative brain weight do not reflect changes in the volume of all brain regions in equal proportions across populations (figure 2).

We found evidence that brain regions are also diverging in association with ecological factors. We found significant differences in the relative volume of all brain regions across environment types (all regions *p* < 0.01, figure 2; electronic supplementary material, figure S8 and table S9) and across populations with varying predation estimates (all regions *p* < 0.0001, electronic supplementary material, table S9). Particularly, the telencephalon and optic tectum are larger in lotic environments (electronic supplementary material, figure S8). For the optic tectum and medulla, we found differences in the degree of sexual dimorphism in the relative volume of these regions across populations (interaction between sex and population: *p* < 0.05; figure 2; electronic supplementary material, table S9), although for the remaining regions this interaction remained borderline significant (*p* < 0.1 figure 2; electronic supplementary material, table S9).

**Figure 3.**
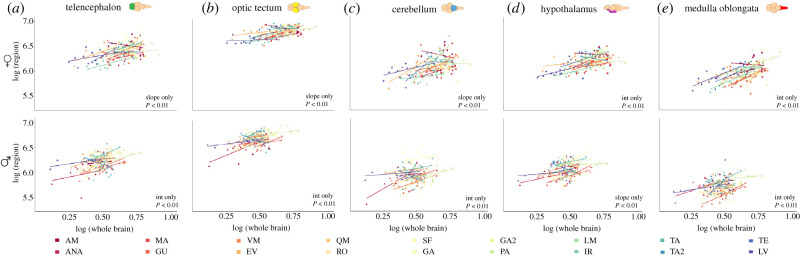
Allometric relationships of each brain region relative to whole brain in females (top row) and males (bottom row) for (*a*) telencephalon, (*b*) optic tectum, (*c*) cerebellum, (*d*) hypothalamus and (*e*) medulla oblongata. Each coloured line corresponds to the regression line of this relationship for each separate population following the colour scheme in legend, with coloured points representing individuals. For each panel, the *x*-axis corresponds to the log-transformed brain weight and the *y*-axis to the log-transformed volume of each brain region. Illustrations correspond to the guppy brain, with the brain regions highlighted in different colours: telencephalon in green, optic tectum in yellow, the hypothalamus in purple, cerebellum in blue and medulla in red. (Online version in colour.)

We further examined the changes in relative brain region volumes relative to the whole brain, testing for differences in the allometric scaling of the different brain regions (figure 3) across populations. Here, we evaluated whether there are significant differences in the slope and/or intercept of the regression between the volume of each brain region and whole brain weight across populations [66] (electronic supplementary material, table S10). In general, finding significant differences in the intercept of this regression across populations indicates that the region concerned grows at the same rate relative to brain weight but differs in relative volume across all populations. On the other hand, finding significant differences in slope for the regression across populations indicates that the concerned brain region does not increase at the same rate relative to brain weight across populations. In females, the most parsimonious model according to AIC for telencephalon, optic tectum and cerebellum is one in which the slopes of the relationship between these brain regions and brain weight differ across populations. On the other hand, for the medulla and the hypothalamus, the most parsimonious model is one in which the intercepts of the regression differ across populations (figure 3). For males, the best fit for the hypothalamus was a model indicating differences in the slope, while for other brain regions, the model with the best fit was one that allows for differences in the intercept of the allometric regression across populations (figure 3; electronic supplementary material, table S10).

### (c) Relationship between neuroanatomy and sexual traits

We found ample variation for all measured sexual traits across populations as shown in the electronic supplementary material, figure S9. An initial multivariate analysis revealed the sexual traits we examined are varying along three main principal components that explain 72% of the variation (electronic supplementary material, figure S10). In summary, the principal component analysis (PCA) revealed tail length and the area of black patches are highly correlated, while the gonopodium length, area of orange and the saturation of these patches covary along a separate axis (electronic supplementary material, figure S10).

**Figure 4.**
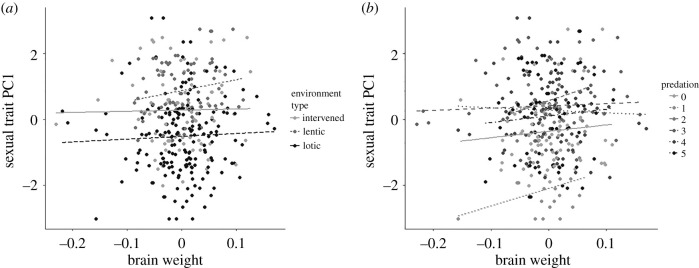
Relationship between sexual traits PC1 (summarizing variation in area of black patches and tail length) and relative brain weight in (*a*) different environment types and (*b*) in different predation regimes. Here we used residuals from a linear model testing the association between sexual trait PC1, brain weight and the various environmental variables examined, including body length as a covariate.

We used linear models in order to test whether sexual traits, more specifically the three first sexual traits principal components, are associated with relative brain weight and the various environmental variables we examined. The first sexual trait PC model revealed a significant interaction between brain weight and environment type (*F* = 3.451; *p* < 0.05, electronic supplementary material, table S11), as well as an interaction between these factors and predation (*F* = 2.836, *p* < 0.05, electronic supplementary material, table S11). Since the first sexual trait PC reflects variation in tail length and the area of black patches, this result indicates the relationship between these sexual traits and brain weight is significant but changes across populations in varying environment types and predation risks (figure 4). We found no significant associations between sexual trait PCs and the volume of any of the brain regions (electronic supplementary material, table S12).

## 4. Discussion

We found pervasive variation in relative brain weight and brain region volumes across populations, likely in response to diverse environmental pressures and cognitive demands across sampling sites. In teleosts, the skull does not constrain the growth of the brain [18,49] allowing the relative size of different brain regions to reflect to the functional abilities necessary to adapt to different environments [17,23,68–70]. The volumes of brain regions have been related to ecological factors that impact fitness, such as habitat complexity, social factors and predation in various taxa [18,22,70,71].

### (a) Brain divergence in different types of environments

The variation we found in relative brain weight and brain region volumes is related to each population's type of environment. It is noteworthy that guppies in lotic environments have a larger optic tectum and telencephalon, regions involved in visual processing, decision making/cognitive processes and spatial memory [69], which can be critical for foraging. In rivers and streams (lotic), water currents and rocks create a complex environment, with more heterogenicity and diversity [72–74], than the more static lentic environments [75,76]. Our results are therefore consistent with the general prediction more demanding habitats that require greater cognitive and sensory abilities for survival [77–80], will select for larger brains particularly, larger dorsal areas of the brain [21,49].

### (b) Predation is associated with brain weight and neuroanatomy

Predation can have a significant impact on brain evolution, but the direction of this relationship is not always the same [25,68,81,82]. Here, we find guppies from natural populations with higher predation risk tend to have larger brains. Our findings are in line with previous reports [26] suggesting predation favours larger brains and drives differences in neuroanatomy by imposing selective pressure for cognitive and sensory processes that benefit predator evasion. We also found this relationship is sex-dependent and present in both sexes, unlike previous studies in which this effect is stronger or limited to male guppies [25], which are more vulnerable to predation [28]. Further studies are necessary using more detailed predation data in both sexes to better understand how predation risk shapes brain neuroanatomy in this system.

### (c) Allometric scaling of the brain changes across populations

Our evaluation of the allometric relationship between the volume of each brain region and whole brain weight can contribute to the existing debate between the concerted [39–41] and mosaic models of brain evolution [35–37]. We found brain regions are not growing with the same scaling relative to the whole brain across all studied populations. Even if it is still likely that the proportions of different brain regions are under some degree of constraint, we still find evidence that different brain regions are growing independently favouring the ‘mosaic’ evolution hypothesis. The independent development of the different brain regions allows for the evolution of volumetric differences across populations because of plasticity or adaptation.

Our results can be directly compared to previous work in which laboratory selection lines that differ drastically in brain size, but raised under the same environmental conditions were shown to maintain the same brain region proportions [38,49]. These contrasting results suggest the functional demands that accompany variation in the environment across populations could contribute to the neuroanatomical divergence we observe. Nevertheless, further work is necessary to link specific environmental factors to the neuroanatomical variation in this and other wild systems.

### (d) No association between brain weight and Ang-1 expression

Variation in the expression of the gene Angiopoietin-1 (*Ang-1*), a regulator of angiogenesis, was shown to be at the basis of brain size differences in laboratory guppies and zebrafish [67]. We measured the relative expression of *Ang-1* across selected populations in our system, predicting its expression would be correlated with brain size across populations, but found no evidence for this association. There are multiple explanations for this: it is possible either noise in data from wild individuals, or a more complex genetic architecture underlying brain size evolution in the wild could be obscuring a possible association [67]. Moreover, even if *Ang-1* levels in the brain of adults were shown to be associated with brain size, *Ang-1* expression during development could also play a crucial role determining brain size. Our data is too limited to draw definitive conclusions beyond the preliminary observation that *Ang-1* relative expression in adults does not seem to be associated with relative brain size in the four examined populations.

### (e) Sexual dimorphism in brain weight and neuroanatomy

As predicted, we found varying degrees of sexual dimorphism in relative brain size and the relative brain region volumes across wild populations. These differences are probably a reflection of the well-documented differences in selection pressures affecting each sex [83–86], which can lead to divergent cognitive and functional demands between sexes [31–34]. Finding that the optic tectum exhibits male-biased sexual dimorphism in most populations is consistent with males' increased predation vulnerability and previous reports that males develop larger brains in the presence of predators [25]. The optic tectum has a crucial role in motion detection and fleeing response [87], and has been associated with foraging strategies and the presence of predators with active hunting strategies [18,49]. This hypothesis is further supported by the lack of dimorphism in relative optic tectum volume in intervened environments, which are particularly poor in predators. Females, on the other hand, are more innovative and efficient foragers and tend to be more active than males [88,89]. These increased cognitive demands in females could contribute to the observed female-biased sexual dimorphism in the relative volume of the telencephalon and cerebellum, brain regions with a proven role in cognitive abilities [90,91].

### (f) Association between sexual traits and brain weight in the wild

This comparative study across natural populations allowed us to investigate the relationship between sexual traits and brain size, testing hypotheses on the role of sexual selection in brain evolution. As predicted by hypotheses proposing a positive association between brain size and sexual traits due to the higher cognitive demands of mating [54–57], we found an association between a sexual trait PC summarizing variation in the area of black patches and tail length, and brain weight. In the wild, however, this relationship seems to change across environment types and with exposure to different predation pressures. Also, in contrast with previous studies [50,53], we do not find evidence that this association exists for all sexual traits. It is possible that the positive correlation reported between other sexual traits and brain weight in the laboratory could only be evidenced in the absence of environmental variation. Our data suggest environmental pressures are strong drivers of brain divergence even if other factors, such as mating behaviour, could be at the base of our results.

Here, we were able to evaluate multiple hypotheses on brain evolution. Our findings complement and extend previous laboratory studies in guppies, allowing us to investigate the importance of ecological and sexual pressures on brain differentiation. While laboratory studies allowed us to clearly understand the relationship between brain size, neuroanatomy, cognition and many physiological traits, our findings suggest these relationships change in the face of the demands imposed by different environments in the wild.

## Data Availability

The data are provided in the electronic supplementary material [92].
